# Evaluating the User Experience of a Smartphone-Delivered Sexual Health Promotion Program for Older Adults in the Netherlands: Single-Arm Pilot Study

**DOI:** 10.2196/56206

**Published:** 2024-04-03

**Authors:** Ana Correia de Barros, Mariëtte Bergmans, Kreshnik Hasanaj, Drianë Krasniqi, Catarina Nóbrega, Bruna Carvalho Carneiro, Priscila A Vasconcelos, Ana Luísa Quinta-Gomes, Pedro J Nobre, Joana Couto da Silva, Cristina Mendes-Santos

**Affiliations:** 1 Fraunhofer Portugal AICOS Porto Portugal; 2 PCOB Utrecht Netherlands; 3 SPRIGS RND Pristina Kosovo; 4 Center for Psychology, Faculty of Psychology and Education Sciences, University of Porto Porto Portugal

**Keywords:** internet interventions, mobile health, mHealth, older adults, sexual health, smartphone, user experience, pilot study, mobile phone

## Abstract

**Background:**

Sexual health is an important component of quality of life in older adults. However, older adults often face barriers to attaining a fulfilling sexual life because of issues such as stigma, lack of information, or difficult access to adequate support.

**Objective:**

We aimed to evaluate the user experience of a self-guided, smartphone-delivered program to promote sexual health among older adults.

**Methods:**

The mobile app was made available to community-dwelling older adults in the Netherlands, who freely used the app for 8 weeks. User experience and its respective components were assessed using self-developed questionnaires, the System Usability Scale, and semistructured interviews. Quantitative and qualitative data were descriptively and thematically analyzed, respectively.

**Results:**

In total, 15 participants (mean age 71.7, SD 9.5 years) completed the trial. Participants showed a neutral to positive stance regarding the mobile app’s usefulness and ease of use. Usability was assessed as “Ok/Fair.” The participants felt confident about using the mobile app. To increase user experience, participants offered suggestions to improve content and interaction, including access to specialized sexual health services.

**Conclusions:**

The sexual health promotion program delivered through a smartphone in a self-guided mode was usable. Participants’ perception is that improvements to user experience, namely in content and interaction, as well as connection to external services, will likely improve usefulness and acceptance.

## Introduction

### Background

Sexual health is a component of general health [1] and quality of life in older age [2]. However, older age is also associated with barriers to a fulfilling sexual life [3-5]. Many older adults are sexually active [6] but are at a higher risk than the general population to present sexual difficulties and dysfunctions. Older women often report decreased libido or lack of vaginal lubrication, whereas erection issues, reduced sexual desire, or being unable to reach orgasm are difficulties regularly reported by men [7]. In health care services, sexual difficulties are often untreated [8] and aggravated by poor communication related to lack of appropriate and case-specific information, lack of training among clinicians, or negative social beliefs and societal stigma, which makes it difficult for both patients and clinicians to bring about the topic [9]. Therefore, identifying the means of circumventing societal stigma and providing timely and adequate support are 2 important courses of action to promote sexual health among older adults.

As the prevalence of smartphone ownership and access to the internet increase [10], there is an opportunity to use these technologies to deliver ubiquitous sexual health support in an inconspicuous manner, that is, one that does not overly expose support seekers to fear of social judgment. Smartphones, as they are intimate technologies that ubiquitously accompany their owners, seem to be an adequate means for the delivery of sexual health promotion programs. Although there is evidence of the efficacy of internet-based sexual health interventions for sexual dysfunction [11] or sexual health education [12], the literature is nonexistent on smartphone-based sexual health interventions targeting older adults [13].

Critical to the acceptance and adoption of such technologies is the user experience they provide [14,15]. Coined by Don Norman [16], the term “user experience” was used by the author to characterize all the sets of experiences a user has with a product throughout a user journey, from intention to use until postuse reflections [17]. Therefore, the concept goes beyond usability, defined by International Organization for Standardization as “the extent to which a system, product or service can be used by specified users to achieve specified goals with effectiveness, efficiency and satisfaction in a specified context of use” [18]. Designing positive user experiences with mobile digital technologies for older adult users has been a focus of many studies because the levels of engagement have been low, thus hampering the potential health benefits of such technologies [19]. Research has found that older adults’ user experience with mobile digital health could be improved if the technology considered potential user sensorimotor and cognitive issues, users’ motivation, and social support [19] as well as if it promoted more personalized experiences and trust [20]. Although there are general guidelines on designing for accessibility and inclusive design [21,22], best practices for designing digital technologies for sensitive topics such as sexuality and intimacy are lacking [23]. Understanding older adults’ experiences with such technologies in the topic of sexual health is critical to improving their acceptability, usability, and adoption, so that they can deliver positive outcomes. However, no study has yet reported on older adults’ user experiences with smartphone-delivered sexual health promotion programs.

To address these gaps, we have designed a smartphone-based sexual health promotion program [24] under a European project called Anathema (reference AAL-2020-7-133-CP). This program was made available to older adults in a longitudinal study during which we assessed the participants’ user experience with the software. The findings contribute to the body of knowledge on older adults’ preferences, use, and appropriation of digital technologies for sexual health and the design of smartphone-based sexual health promotion programs targeting this population.

### Aim

The aim of this study was to evaluate the user experience of Anathema, a self-guided, smartphone-delivered program to promote sexual health among older adults.

### Anathema Mobile App Overview

The mobile app used in this study was developed using a participatory design approach [25], which involved users from 3 European countries using the following methods: questionnaires, interviews, focus groups, usability tests, and co-design workshops [23,26].

The app is available for Android and iOS operating systems and contains a sexual health promotion program tailored to older adults. The program, which has an 8-week duration, is organized into 5 modules (which include chapters and subchapters):

- *Module 1—Let’s talk about sexuality (week 1)*: features information on male and female anatomies, sexual response, the importance of sexual pleasure, and sexual rights.
- *Module 2—When age and illness come in the way (week 2)*: addresses successful aging; the physiological, cognitive, and emotional changes in older age; and the main sexual problems and sexual dysfunction in older age.
- *Module 3—Emotional and physical intimacy (weeks 3-6)*: covers psychoeducation on the cognitive behavioral therapy model and the impact of sexual beliefs, thoughts, and emotions on sexuality. It includes exercises for cognitive restructuring, mindfulness, and communication skills training.
- *Module 4—Exploring one’s sexuality (week 7)*: delivers information on sex aids and strategies to enhance sexual pleasure and satisfaction and includes sexual skills training and mindfulness exercises.
- *Module 5—Planning for a long-term fulfilling sex life (week 8)*: targets on relapse prevention with a focus on strategies to maintain progress and prevent setbacks. It also shares strategies to promote a healthy lifestyle and sexual health.

**Figure 1:**
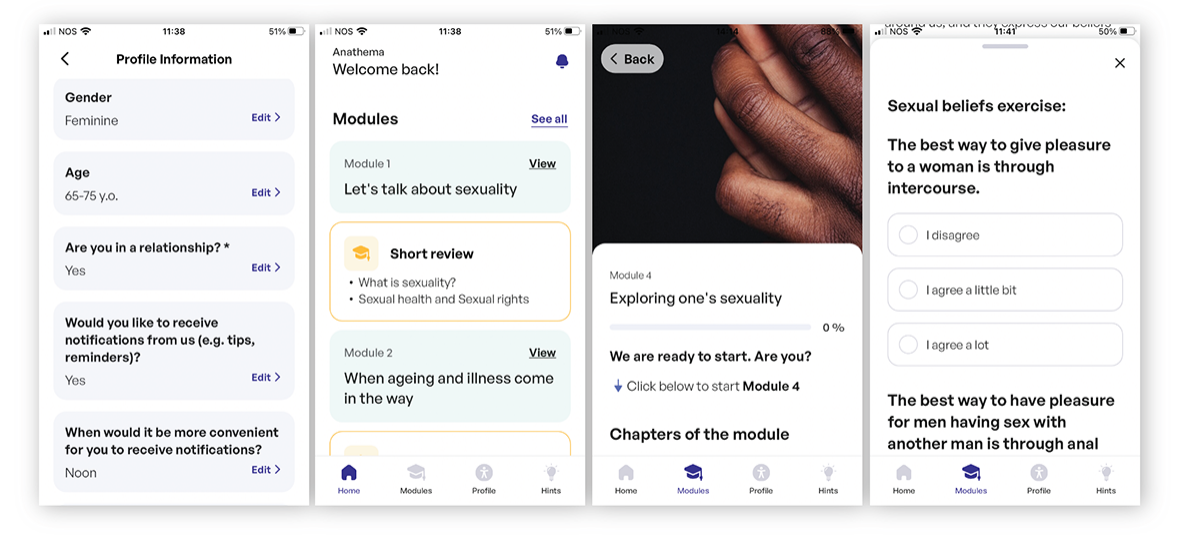
Sample screenshots from the Anathema app (left to right): personal information, overview of modules, introduction to module, exercise.

Each module is unlocked upon the completion of the previous module to ensure knowledge and skills acquisition. The chapters and subchapters are made of content in the form of text, images, and videos. The program also includes exercises such as written reflections or answers to multiple-choice questions using radio buttons (Figure 1). The app is available in English, European Portuguese, German, and Dutch languages.

The mobile app performs passive data collection through timestamp logs of interactions (eg, module completion date) as well as active data collection through logs of users’ inputs on exercises. Another tool, Trial Monitor [27], fetches data from the database and shows visualizations thereof to the research or therapist teams.

## Methods

### Study Design

The study design was a single-arm pilot study with older adults (aged ≥55 years) testing the self-guided format of the sexual health promotion program in its Dutch version. The pilot study was conducted to assess user experience of the program. The content, structure, and format were also preliminarily evaluated toward the identification of improvements to the program and technological means of its delivery.

### Inclusion and Exclusion Criteria

The inclusion criteria for participation in this trial were as follows: (1) being able to provide informed consent, (2) being aged ≥55 years, and (3) having digital skills and internet access. The exclusion criteria were as follows: (1) having a severe psychiatric disorder or alcohol or substance abuse; (2) taking medication that could interfere with sexual response; (3) having an uncontrolled medical condition that could interfere with sexual health; and (4) currently being on psychotherapy for sexual or intimate problems or for other psychological problems or current participation in another intervention study or clinical trial (or both).

### Study Procedures

In a previous phase of this research, 1119 older adults, recruited through the contact list of the Dutch senior organization Katholieke Bond van Ouderen - Protestant Christelijke Ouderen Bond (KBO-PCOB), answered a questionnaire on unmet sexual needs [26]. In this questionnaire, the respondents were asked to indicate whether they would be available for future research within the same research project. Respondents who gave a positive reply were regularly invited to participate in user research activities throughout the research project [23], including the pilot study described in this paper. The majority of this subsample (N=346) were men (69.4%), had a high education level (53.2%), and were retired (89.9%). For the pilot study, further potential participants were contacted via other KBO-PCOB channels, including KBO-PCOB’s employees.

Upon signing the informed consent form, participants were asked to complete a web-based screening questionnaire. If deemed eligible to participate in the study, the participants were asked to answer a web-based sociodemographic questionnaire. After completing the questionnaire, participants were provided access to the Anathema app and were prompted to complete the program in 8 weeks.

Once they had completed the 5 modules in the app, participants were asked to fill in a web-based, self-developed user experience questionnaire (Multimedia Appendix 1), which also included the System Usability Scale (SUS). Participants were then invited to participate in a semistructured debriefing interview about (Multimedia Appendix 2) their experiences with the program.

### Metrics and Data Analyses

The main outcome of the study was user experience, which included dimensions of usefulness and usability. User experience was assessed after the intervention with a self-developed multiple-choice list of characteristics (answer options: *accessible*, *arousing curiosity*, *attractive*, *boring*, *elegant*, *fascinating*, *helpful*, *instructive*, *meets expectations*, and *strenuous*), a question on free grading of the app from 1 to 10, with 10 being the highest grade, a Net Promoter Score question (answer options: *Yes*/*No*/*Don’t know*), and a semistructured debrief interview with questions addressing usefulness, usability, feasibility, clinical aspects, and implementation (Multimedia Appendix 2). Perceived usefulness was assessed using a self-developed 5-point Likert scale assessing the program in general, each module, and exercises. Usability was assessed using a self-developed 5-point Likert scale on perceived ease of use and perceived readability, as well as with the Dutch version of the SUS [28]. Assessment of the self-perceived contribution of the program to changes in satisfaction and pleasure in sex life was also performed postintervention with a single-item question (4-item descriptive rating scale).

To characterize the study sample, sociodemographic variables were collected using a self-developed questionnaire assessing age, education, professional status, gender, sexual orientation, marital status, current sexual partnership status, satisfaction with current sex life (5-point Likert scale), self-rated quality of life, and degree of satisfaction with their own health (based on items 1 and 2 from World Health Organization Quality of Life Brief Version [29]).

The interviews were audio-recorded and partially transcribed for relevant content. The transcriptions, written in Dutch, were then translated into English by a native Dutch speaker (MB) for analysis by a non-Dutch speaker (ACB). The questionnaire and the interview data were analyzed descriptively and thematically, respectively.

### Ethical Considerations

The study was approved by the Ethics Committee of the Faculty of Psychology and Educational Sciences, University of Porto (reference 2022/01-05b). All potential participants were informed about the study objectives and procedures. The participants who agreed to participate signed the informed consent form. There was no compensation or payment offered to the participants.

## Results

### Participants

A total of 400 participants were approached to participate in this study. Most participants did not provide a reason for declining or not answering the invitation. Among those who did (n=47), the reasons given were that participants were no longer interested (n=15), considered the pilot required too much commitment or effort (n=12), felt uncomfortable with the topic (n=9), considered they did not meet the criteria (n=5), or had a malfunctioning email (n=5). We also received information that one person had died.

In total, 23 participants agreed to participate and completed a web-based screening questionnaire to confirm the eligibility criteria. All participants were deemed eligible and were given access to Anathema after answering a sociodemographic questionnaire.

A total of 8 participants dropped out of the study. Of them, 4 participants did not provide any reasons for abandoning the study. Those who did shared the following reasons: discontinued access to the internet (n=1), dissatisfaction with the fact that future content modules were locked (n=1), inability to install and open the app (n=1), and lost motivation to use the app (n=1). A total of 15 participants used the Anathema app, having completed all the modules and completed the final questionnaire on user experience and usability. In total, 8 participants agreed to participate in a debriefing interview.

The 15 participants who used the app and answered the final questionnaire were 7 cisgender women and 8 cisgender men aged between 56 and 85 years (mean 68.3, SD 9.5 years). Most (n=12) were retired, and most (n=10) had completed higher professional education. Overall, 6 participants were married, 4 were single, 3 were cohabiting, and 2 were widowed (Table 1).

**Table 1:** Sociodemographic characteristics of the sample (N=15).

Characteristics		Values
**Gender, n (%)**		
	Female	7 (47)
	Male	8 (53)
**Marital status, n (%)**		
	Single	4 (27)
	Cohabiting	3 (20)
	Married	6 (40)
	Widowed	2 (13)
**Professional status, n (%)**		
	Employed	3 (20)
	Retired	12 (80)
**Education, n (%)**		
	Secondary professional education	2 (13)
	Higher professional education	10 (67)
	University or scientific training	3 (20)
Age (years), mean (SD; range)		71.7 (9.5; 56-85)

Most of the 15 participants were exclusively heterosexual (n=12), most had sex with a partner in the context of an exclusive relationship with that person (n=11), and the level of sexual satisfaction was heterogeneously distributed, as shown, together with complete sexual characteristics (Table 2). The sample comprised participants who tended to positively rate their quality of life and health (Table 3).

**Table 2:** Sexual characteristics of the sample (N=15).

Characteristics			Baseline, n (%)
**Sexual orientation or preference**			
	Exclusively heterosexual	12 (80)	
	Mainly heterosexual	2 (13)	
	Exclusively homosexual	1 (7)	
**Current sexual partners**			
	Sex with a partner, in the context of my exclusive relationship with him or her	11 (73)	
	Casual sex with a partner	1 (7)	
	No sexual partner	3 (20)	
**Satisfaction with current sex life**			
	Very satisfied	3 (20)	
	Satisfied	5 (33)	
	Neither satisfied nor dissatisfied	4 (27)	
	Dissatisfied	3 (20)	

**Table 3:** Perceived quality of life and health satisfaction (N=15).

		Baseline, n (%)	After the test, n (%)
**Rating of quality of life^a^**			
	Very good	8 (53)	9 (60)
	Fairly good	7 (47)	5 (33)
	Neither good nor bad	—^b^	1 (7)
**Satisfaction with health^c^**			
	Very satisfied	8 (53)	7 (47)
	Satisfied	7 (47)	7 (47)
	Neither satisfied nor dissatisfied	—	1 (7)

^a^Original wording: How would you rate your quality of life? Responses were rated on a 5-point Likert scale: 1=very bad to 5=very good.

^b^Not available.

^c^Original wording: How satisfied are you with your health? Responses were rated on a 5-point Likert scale: 1=very dissatisfied to 5=very satisfied.

### User Experience

In this section, we present the quantitative and qualitative results of the participants’ user experience (Table 4). As we do so, we provide interpretations of the results mostly because of the interpretation required by the analysis of the interview data. Therefore, we discuss some of the results as we present them.

**Table 4:** Results of the user experience questionnaire (N=15).

			Values
**Would recommend Anathema to friends or family (net promoter score)^a^, n (%)**			
	Yes	6 (40)	
	No	6 (40)	
	Doesn’t know	3 (20)	
**Perceived usefulness of app^b^, n (%)**			
	Very useful	2 (13)	
	Useful	6 (40)	
	Neither useful nor useless	6 (40)	
	Useless	0 (0)	
	Extremely useless	1 (7)	
**Perceived usefulness of exercises^c^, n (%)**			
	Very useful	0 (0)	
	Useful	7 (47)	
	Neither useful nor useless	3 (20)	
	Useless	3 (20)	
	Extremely useless	2 (13)	
**Perceived ease of use^d^, n (%)**			
	Very easy	1 (7)	
	Easy	7 (47)	
	Neither easy nor difficult	5 (33)	
	Difficult	2 (13)	
**Readability^e^, n (%)**			
	Very easy	1 (7)	
	Easy	7 (47)	
	Neither easy nor difficult	5 (33)	
	Difficult	2 (13)	
System Usability Scale score, mean (SD; range)			56.3 (19.1; 20-85)
Score (1-10) given to Anathema app, mean (SD)			6.5 (1.8; 2-9)
**Perceived impact of Anathema app in satisfaction and pleasure^f^, n (%)**			
	Positive impact	4 (27)	
	No change	7 (47)	
	Negative impact	1 (7)	
	Doesn’t know	3 (20)	

^a^Original wording: Would you recommend the Anathema app to friends and/or family members?

^b^Original wording: How useful do you think the Anathema app is for older adults? Responses were rated on a 5-point Likert scale: 1=extremely useless to 5=very useful.

^c^Original wording: How useful did you find the (writing) exercises you were offered? Responses were rated on a 5-point Likert scale: 1=extremely useless to 5=very useful.

^d^Original wording: How easy was it for you to use the Anathema app without any help from others? Responses were rated on a 5-point Likert scale: 1=extremely difficult to 5=very easy.

^e^Original wording: How readable did you find the content of the Anathema app? Responses were rated on a 5-point Likert scale: 1=extremely difficult to 5=very easy.

^f^Original wording: Do you have the impression that the Anathema app can help you change satisfaction and pleasure in your sex life? Rated using a descriptive scale: Don’t know; No, no change; Yes, namely less satisfying and fun; Yes, namely more satisfying and fun.

**Figure 2:**
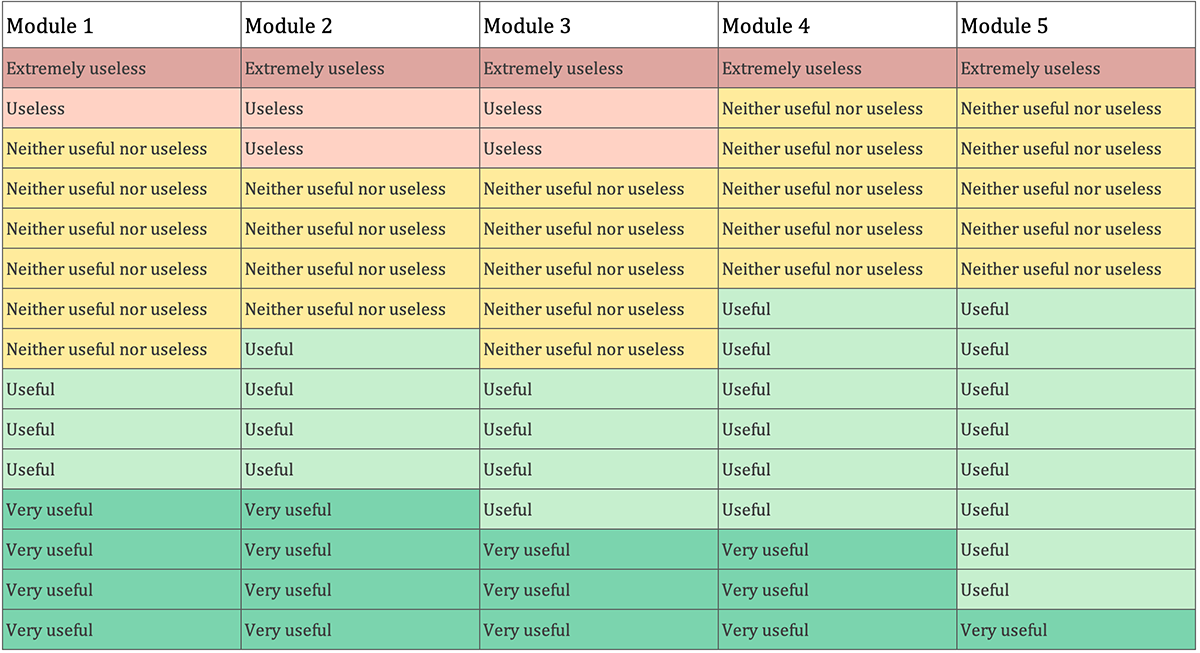
Visualization of perceived usefulness by module.

Most participants showed a neutral to positive stance toward the app regarding its *usefulness*. There are some nuances when analyzing the perceived usefulness per module, as illustrated in Figure 2. Modules 2 and 3 had slightly more polarized responses. Modules 1 and 2 were found to be “very useful” for more participants, likely because of the reasons given in the interviews: participants learned new concepts, learned to understand what is normal in aging (“I end up thinking about the part about body ageing. That’s reliable information that I can’t easily get anywhere else today” [P03]), were made to rethink the way in which they faced sexuality, and also learned about the genitalia of other sexes:

Nice to read some details about genitals[...] also from the opposite sex, how something works.

P11

Enlightening. I did benefit from seeing what a prostate looked like.

P04

Other highlighted learning points from the program are the importance of communication and the fact that sexuality does not need to be equated with penetration. Something some participants missed was the possibility to ask the app questions about their specific problems, ask questions anonymously, or to be able to search for certain themes that could be of more interest to them.

Seven participants evaluated the *exercises* as useful, whereas the other 8 found them neutral (n=3), useless (n=3), or extremely useless (n=2). Crossing these results with the information provided in the interviews, one can infer that there were 2 aspects that hindered the experience with the exercises: on the one hand, participants struggled with long text input on their smartphone keyboards; on the other hand, for this group, the feeling of being “schooled” by the app was not equated with positive emotions, thus negatively impacting the experience. Finally, in the interviews, participants revealed that some exercises helped them think of sexuality in a different way, which they experienced as being positive.

**Figure 3:**
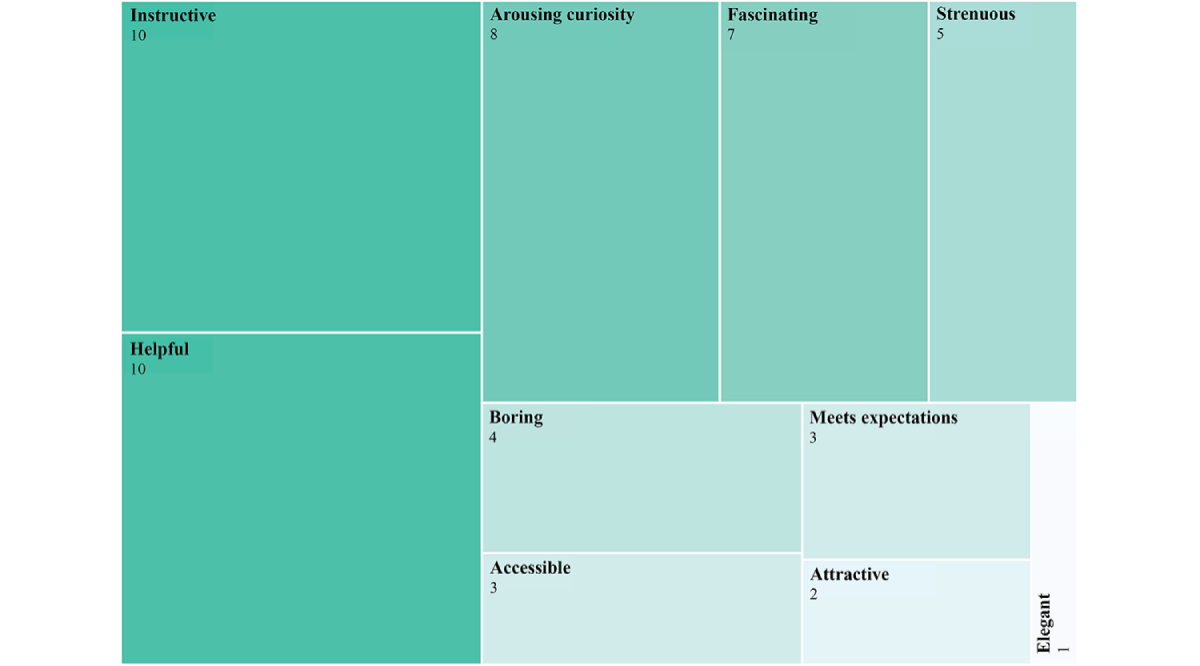
Visualization of qualifiers attributed to the Anathema app and how often they were attributed.

When asked to attribute *characteristics* to the *Anathema app*, most participants selected a set of descriptors displayed in Figure 3, but the number of choices varied from a single adjective to 6 adjectives. Most of the qualifiers have positive valence, with the exception of “boring” and “strenuous,” with 4 and 5 mentions, respectively. In line with the data collected through the interviews, the participants perceived that they had learned from the app. However, only 5 participants assessed the app as having the potential to help change their sexual satisfaction and pleasure.

The interviews also revealed that participants appreciated the app aesthetically, which connects to the descriptors that were chosen, as well as the tone of voice that was adopted for the content, which, in some cases, helped them deal with a sensitive topic:

I admire that this can be done in an app. Good looking and doesn’t scare someone. I managed to deal with such a sensitive topic. [It’s] friendly and nicely constructed.

P22

For 2 participants, the communication style options were not the most appropriate, for example, when showing an animated video of an anthropomorphized clitoris. Although the photographs were selected based on a survey conducted by the research team about the characteristics of photos that were appreciated by Dutch older adults, 2 interviewees did not find them totally appropriate, for example, some having a comical or childish tone, representing too young people, or not representing enough diversity.

Taking the *net promotor score* as an indicator of satisfaction, we can see that opinions were divided. Three participants did not know whether they would recommend the app to friends or family, whereas the remaining 12 participants were equally divided between wanting to recommend and not wanting to do so. In the debriefing section of the interviews, participants who were not certain whether to recommend Anathema expanded on this. They explained that they think the app has potential but that it needs certain improvements, as described earlier, for them to confidently recommend it to others.

The average SUS score, which measures *usability*, stood at 56.3, which, according to the scoring standards, corresponds to an assessment of “OK/Fair” [30]. Based on the averages per item, we can see that participants tend to think that they do not need help in using the system, although usability is not perceived to be at the excellent level. The level of confidence felt by participants while operating the app was high. Participants generally showed a neutral to positive stance toward the app regarding its *ease of use* and its *readability*. Although most people did not experience trouble reading because of font size or contrast, this was an issue for one of the participants who dropped out:

I also found the fine print difficult. They are clear but with deteriorating eyes good reading requires more effort.

P04

The interviews revealed that the app worked well on participants’ phones and that they found it very convenient. However, participants often wished that the app would also be easy to use on a tablet device or desktop:

Excellent [the experience of using the app on the phone]. Preferably on an iPad, because of the larger screen. On the phone it worked. The smaller keyboard asked more caution, but [it] went fine.

P11

**Figure 4:**
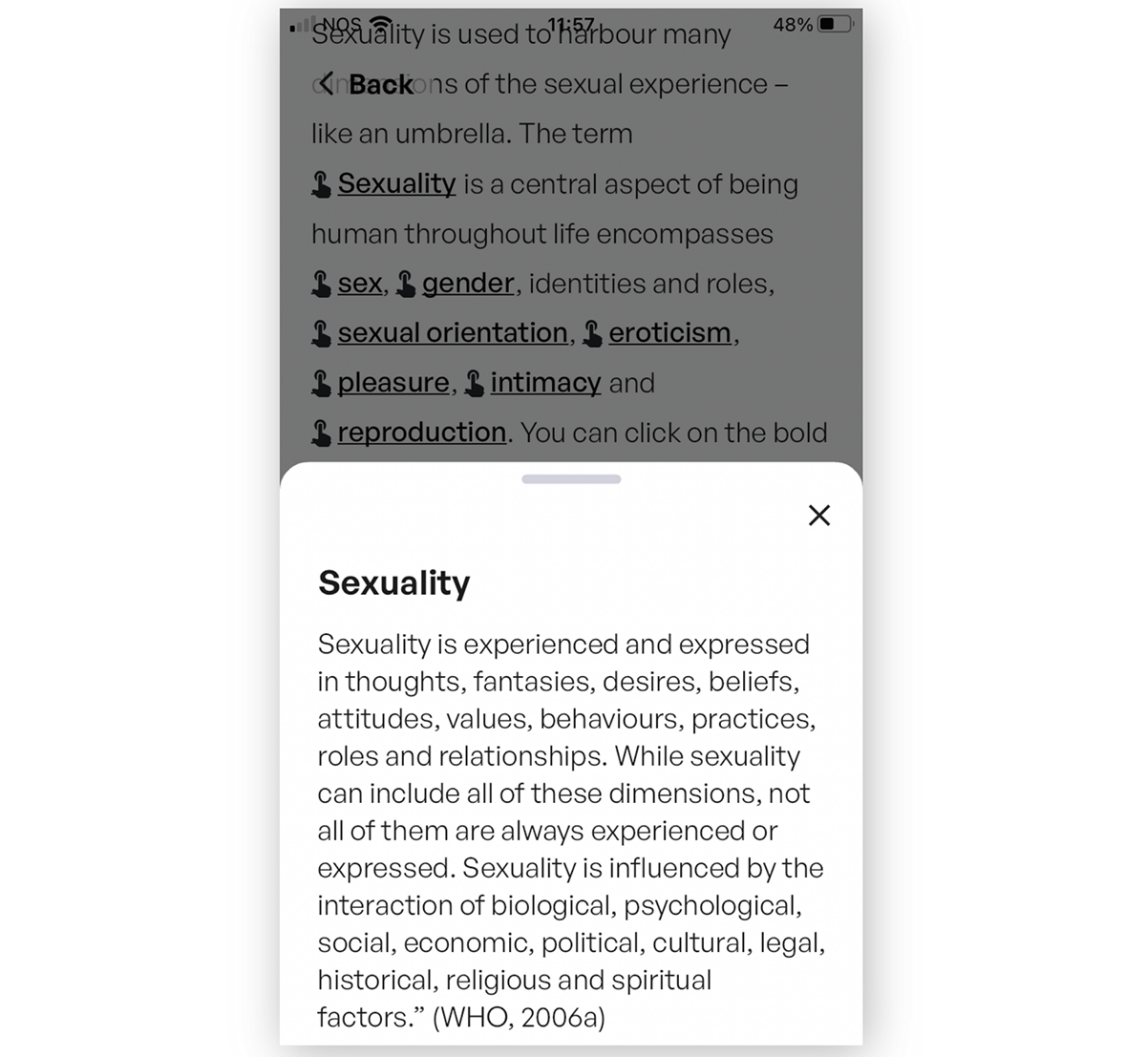
Screenshot from the definition of sexuality, which appears in a slide-up pop-up when the user taps the word “Sexuality” shown in the gray-faded part of the image. WHO: World Health Organization.

With the exception of 2 participants, who suggested direct speech, easier wording, and shorter sentences, interviewees found the wording easy to understand. In total, 2 participants reflected on whether the scientific explanation should be highlighted as is (Figure 4), for instance, on starting the first module with the definition of sexuality or whether it should be made more digestible to engage readers.

Another aspect of readability that was touched on was finding one’s place in the content structure. For 2 participants, it was hard to understand at which stage they were in navigating the app, originating the feeling of being lost: “In a book you can browse through that and then you see where you are. In the app this overview is not so clear” (P06). For those who felt lost, as well as for one participant who would like to revisit specific parts of the content, a possible solution was provided by one participant who said that they missed a way to bookmark “Favorites.”

The participants *appropriated* the app in different ways. There were reports of people using the app only randomly when they found the time, defining a fixed schedule (eg, evening, late at night), or defining a place to use the app (eg, kitchen, home). Common to the participants was the need to use the app alone and undisturbed.

One participant asked the partner to also go through the app, but they did not want it because it was a taboo topic. Three mentioned how they talked to friends or their partners later about the app and what they had learned, for example:

Through the app I can easily talk with my partner about sexuality. The participation, together with my partner, in the previous workshops for Anathema, also contributed to this. The openness of other participants was a good example for me.

P04

While 5 participants stated that there would be no place or time when they felt uncomfortable using the app, the remaining 3 gave some indications thereof. For these participants, it would be important to use the app alone and in a private place. Participants also reflected on how they would like to discuss what they were learning in the app with others but found it stigmatizing:

When I talk to friends about food, for example, all the experiences can be discussed. Apparently, that is not possible when talking about sex.

P20

When I try to discuss with seniors of an association with a Catholic background that I am participating in this project, the reaction is that does not suit our people.

P18

Half of the participants found an *8-week period* to be too long, whereas the other half felt it was an acceptable or good duration. However, participants struggled with the idea of this being time bound in some way because they could not understand why this specific duration was chosen. In 1 case, the participant felt that this indication of duration could send the wrong message: “I have to be ready in 8 weeks” (P03).

Being presented with *content that had a specific reading order* was cumbersome to some participants. This was because, on the one hand, they could feel schooled, and, on the other hand, they did not want to feel that they were losing time in content that they were not interested in. One participant shared their technique for when something like this happened: they just scrolled the content very quickly to get to the bottom and move to the next chapter. Despite negative comments about the locked content (eg, “I wanted to look at a topic in Module 4. But didn’t do that out of irritation at the locks in the extended Module 3” [P04]), participants generally agreed that the content is well structured, being easy to follow. Mindfulness is something that some interviewees found unnecessary. On the other hand, some interviewees would expect to read more about love and affection. There were also other suggestions of curated lists of contacts for further support (eg, participants stated they would like to be able to ask questions to therapists over email) and fitness exercises (eg, pelvic floor muscle exercises).

Although 9 of 15 participants in the questionnaire assessed module 5 as useful or very useful, the interviews revealed a slightly different picture. The interviewees had mixed opinions regarding the usefulness of the last module. With the exception of 1 person, those who found it useful as a recap also reflected on the possibilities of *coupling the app* with curated contacts to therapists to continue exploring the topic or to find tailored help to a specific issue. One interviewee thought about accompanying the app with television or radio shows, stating that this was the reason why they bought a book on sexuality. Another possible extension would be a course, workshops, or group activities that would let people discuss and further explore what they had learned and experienced:

In addition to using the app, it could be interesting to be in a discussion group with other couples as a couple. That could help improve communication about sex. The app provides plenty of conversation material for that.

P11

Interviewees had mixed opinions regarding *whether the app should be paid.* On the one hand, participants shared that they are not used to paying for apps, but on the other hand, they recognized that they might pay for extra services (eg, consultations) and that free apps do not have as much credibility. Credibility is something that participants cling to when reflecting with the interviewer about how to make the app available to more people. Participants concluded that the app could be credibly made available through medical doctors, therapists, or reliable associations. Although this was not asked, participants also shared ideas on how to raise awareness about the Anathema app, for example, through advertisement, television or radio shows, or leaflets.

## Discussion

### Principal Findings

The pilot study conducted in the Netherlands with a group of 15 community-dwelling older adults was a novel study in the field of mobile health apps in sexual health. Although the dropout rate was high (65%), no participants were lost to follow-up or nonuse cases, that is, participants answering the questionnaires without having used the mobile app until the end. We found that the app was usable, that participants showed high levels of self-confidence in using it, that the smartphone can be a useful and private way to have access to reliable sexual health information, that participants foresee how extra services could help tailor the program to their specific needs, and that certain improvements in content and in interaction are likely to increase user experience for this smartphone-delivered sexual health promotion program.

As with other studies in the literature [20], the user experience was negatively affected by a lack of social support for users’ specific issues. In the interviews, participants gave examples of further content on love and affection, a curated list of resources and fitness exercises that they would like to see, and options to search through the content to get the information they were looking for. The lack of social support, ranging from relatives to professionals, also seems to have negatively affected participants’ user experiences. In their systematic review, van Acker et al [19] noted how social support (ranging from relatives to professionals) was an important factor in user experience. In our study, with the exception of 1 participant who could not convince their sexual partner to also use the app, there were no reports of available or lacking support from relatives, but participants specifically mentioned that professional support would be useful in addition to the existing offer. A nuance with relation to the literature [19] is that participants in our study did not require much professional support to interact with the program, but rather as an extension to it, often to attain the personalization requirement we have just described earlier. Furthermore, the participants struggled with the locked content. Although the tunneling technique has been used to increase engagement with intervention or technology, in our study, it did not seem to have this effect. This is similar to recent findings with an intervention for a younger generation [31].

As noted in the literature [20], trust is also an important dimension in user experience. Although not directly asked about it, our interviewees alluded to the element of credibility regarding willingness to pay, which was considered by Hurmuz et al [20] as a metric of user experience. For the participants in our sample, the channel via which they access the app is an important factor at the time of choosing whether to use and ultimately pay for the app.

As measured by the SUS instrument, self-confidence among the participants in our study was high. This might also have been influenced by the level of education and digital literacy of the sample. The level of education might also explain why the participants often alluded to the experience of “being schooled” as a negative valence. Although the tone of voice for the program regarding visual and written content was co-designed [23], it might not have been implemented properly to eliminate this negative experience. This aspect is further discussed in the “Limitations” section below. On the other hand, some users also reacted negatively to content that seemed “too scientific,” and some commented that some terms might not be easy to understand for the wider population. This is at odds with the higher educational level of this sample, but the explanation for the dislike might be related not to the understandability of the content but rather to a kind of experience that users expect when they are using an app that is related to sexuality.

The participants stated that the topic of sexuality was not embarrassing. However, there were some accounts of users requiring privacy when going through the content, one user whose partner did not want to use the app because of the topic, or users commenting on how they did not feel free or at ease discussing the topic with their peers. Therefore, the topic of taboo still requires further research in terms of how much of a barrier it is to accept and use technology around this topic. Participants’ statements in the interviews suggested that a smartphone-based intervention can bring the advantages of ubiquity, intimacy, and anonymity to an intervention that is likely to elicit stigma in some contexts. The program itself was regarded as a trustworthy source of information that participants think is difficult to find on this topic. On the other hand, it could be coupled with more targeted personal services for users who would like to interact with therapists or even join groups willing to openly discuss topics of sexual health. Future research should study the provision of such discussion groups either in person or through moderated and anonymous forums inside the app.

Our study included participants interested in sexual health. In any case, even within our small sample, we witnessed a wide spectrum regarding taboo. For instance, some participants felt blurring genitalia photographs by default with overlaid text: “Sensitive content. Click to view” was condescending, whereas others felt that suggesting exercises for sexual pleasure was going too far. As with other types of apps targeting older adult users, our study saw a large heterogeneity in user preferences. Even if resources are allowed for the software development team to implement ultrapersonalization, we could place a large burden on users upon onboarding to set up preferences, which, in itself, would have a negative effect on technology acceptance. One way of addressing this could be to create certain user profiles and adapt scaffolding techniques that have been used for usability [32] for the purpose of conspicuousness degrees. Future research should work on this balance between a certain level of tailoring to one’s needs and preferences, with time invested in customizing the app.

### Strengths

This was the first study to evaluate the user experience of a self-guided, smartphone-delivered program to promote sexual health among older adults. The mixed methods approach was a strength of this study in the sense that it provided a rich description of participants’ experiences with the app and the program. Without the interviews, we would hardly have had such detailed information that would indicate how to improve the app and the program, as well as a first understanding of how participants appropriated the app.

Our study did not aim at generalizability but rather at an in-depth understanding of user experience, which justified the emphasis on the qualitative data. Through this approach, we derived actionable insights to improve the content, structure, and format of the program.

Although our study was conducted with a small and specific sample of older adults in the Netherlands, the methodology we used allowed us to unveil nuances that can be useful for researchers to consider when implementing smartphone-based programs for sexual health in different populations: the relevance of social support, the credibility of the program, the opportunities that smartphone-based interventions may bring to sexual health interventions in terms of privacy or convenience, and the variability among program users about what might be considered a taboo and how this might impact users’ preferences, practices, and attitudes toward the programs.

### Limitations

As we conducted a user experience pilot study to obtain in-depth feedback, the results might not reflect the characteristics of the older adult population in the Netherlands. Although further research is needed to reach generalizability, this study constitutes a stepping stone in this journey.

The sample characteristics in our study are its greatest limitation. Only one-fifth of our participants were dissatisfied with the current state of their sex lives, and most considered themselves to be in fairly good or good health, which may not be representative of the older adult population. These characteristics may have biased how participants responded to a sexual health promotion program tailored to help users identify and cope with issues related to their sexual health. Our sample also comprised participants with a high level of education. This might explain why some participants felt schooled, as they were already in possession of information that was provided by the program. As participants have suggested, for a future pilot study, it would be advisable to increase the number and type of channels used for dissemination and recruitment, such as the mainstream media. This would help increase the visibility of Anathema and reduce, if not altogether, prevent, selection bias.

The features implemented on the app responded as much as possible to the user research requirements, but this was not always possible or perhaps implemented at its best. In some cases, there were technical limitations that did not allow their implementation. For instance, the app began to be implemented as web based so that it would also run on desktop browsers if participants preferred, but the identification of a problem in a technical component ahead of the implementation process forced the software development team to develop natively for Android and iOS.

We expected this lack of flexibility in the device type to be a negative aspect for some participants. On the other hand, at least once, the preferences collected from participants in user research studies preceding the pilot study were not aligned with the preferences of the pilot study sample. We describe 2 instances of this problem.

The first example relates to the choice of imagery. To select the photos for the app, we conducted a survey with 111 older adults in the Netherlands, in which we showed 10 different pictures and asked participants to rate the pictures, select their favorites, and justify their choices. The survey revealed that participants preferred uplifting, cheerful, and romantic images of participants who were not young but also not too old. The interviews in the pilot study revealed that, for some participants, these images were not appropriate.

Another example was the language used: a series of tests on the preferred tone of voice were used to create the original content in English [23]. The content was translated into Dutch, which went through content reviews from native speakers with experience in older adult care. Nevertheless, for some participants in the sample, the language was described as “too scientific.” It is also possible that the research team was not able to correctly implement the insights from the user research phase, thus causing a mismatch between the users’ expectations and the implemented app. Further research should revise the feedback from the user research phases and cross it with the results from the pilot study to understand where the app can be improved to meet users’ expectations.

Further research should also focus on interaction and content issues to improve current mobile apps toward improving user experience. In particular, there is a need to understand how to balance the quantity and type of content with an engaging user experience. Once an improvement in user experience has been noted through further formative testing, the pilot should be repeated. As there was a mix of negative and positive comments provided by the participants and because the results from the SUS score are aligned with the comments from the interviews, we do not think that social desirability influenced participants’ answers. However, as social desirability plays an important role in sex research surveys, a future pilot study could include a questionnaire (eg, [33]) to control for this effect. Further research should focus on a larger and more diverse sample regarding sexual satisfaction, health status, and literacy level.

Future pilots should include study designs that enable the collection of fine-grained data about the user experience combined with an assessment of the program’s efficacy in improving sexual health so that the aspects of appropriation and how the app fits into participants’ practices could be better understood and, in turn, inform strategies to improve sexual health outcomes, engagement, and user experience with such an intervention.

### Conclusions and Implications for Design

The mobile app of Anathema with a sexual health promotion program delivered in a self-guided mode to a sample of older adults in the Netherlands was assessed as usable. Most participants tended to assess the app and program as useful, but both the app and the program would benefit from certain improvements, which we group under “content” and “interaction” as possible guidelines.

*Content wise*, readability and engagement can be improved by using plainer language in general, revising sections that sound “too scientific” (eg, definition of sexual health) or too medical (eg, content regarding erectile problems was very focused on the urological aspects). Although for some participants, the content was too long, and participants would also prefer not to have locked content, in which case the length would not be a barrier to engagement. It is clear that participants would appreciate more curated content that would refer them to support the community or to further services.

Regarding *interaction*, there are suggestions to enable searching and asking questions so that the user could be directly guided to the content that is of most interest to them or so that they could center their learning in their own experiences. This implies that content is unlocked by default. Participants would also like to bookmark certain sections and have the means to know where they are in the app. Finally, although participants shared that some exercises made them reflect—they saw this as positive—they struggled with the exercises that involved text input. Therefore, the interaction modes in the exercises can be improved. Although participants state they do not want to be schooled, they highlight “learning” as one of the advantages of using the app. In the future, the Anathema app should meet the goal of teaching without resembling a schoolbook. This was highlighted by participants who were expecting more interactivity from the app rather than an app that reads like a book.

## Appendix

Multimedia Appendix 1 Posttest questionnaire.

Multimedia Appendix 2 Semistructured interview script.

## Abbreviations

KBO-PCOB: Katholieke Bond van Ouderen - Protestant Christelijke Ouderen Bond
SUS: System Usability Scale
