# Supplementary material for: Evaluating the User Experience of a Smartphone-Delivered Sexual Health Promotion Program for Older Adults in the Netherlands: Single-Arm Pilot Study
Source: JMIR Hum Factors. 2024 Apr 3;11:e56206. doi: 10.2196/56206 (PMC11024746; doi:10.2196/56206)
Supplement: Multimedia Appendix 1 [file humanfactors_v11i1e56206_app1.docx]

## Appendix I – Post-test questionnaire

**I – Experience using Anathema app**

1. **How useful do you think the Anathema app is for older adults?**

Select your answer based on the scale below.

Extremely useless

Useless

Neither useful nor useless

Useful

Very useful

1. **How easy was it for you to use the Anathema app without any help from others?**

Select your answer based on the scale below.

Extremely difficult

Difficult

Neither easy nor difficult

Easy

Very easy

1. **How many hours have you used the Anathema app?**

(Add up the time you spent reading and completing the exercises.

If you can't give us the exact time, please give us an estimate.)

|  |
| --- |

1. **How readable did you find the content of the Anathema app?**

Select your answer based on the scale below.

Extremely difficult

Difficult

Neither easy nor difficult

Easy

Very easy

1. **How useful did you find the content of the 5 modules?**

Select your answer based on the scale below

|  | Extremely useless | Useless | Neither useful nor useless | Useful | Very useful |
| --- | --- | --- | --- | --- | --- |
| Module 1 |  |  |  |  |  |
| Module 2 |  |  |  |  |  |
| Module 3 |  |  |  |  |  |
| Module 4 |  |  |  |  |  |
| Module 5 |  |  |  |  |  |

1. **How useful did you find the (writing) exercises you were offered?**

Select your answer based on the scale below

Extremely useless

Useless

Neither useful nor useless

Useful

Very useful

1. **What features do you associate the Anathema app with?**

(Mark all that apply. Multiple answers are possible.)

Instructive

Meets expectations

Arousing curiosity

Boring

Accessible

Attractive

Strenuous

Elegant

Fascinating

Helping

1. **Do you have the impression that the Anathema app can help you change satisfaction and pleasure in your sex life?**

Yes, namely more satisfying and fun

Yes, namely less satisfying and fun

No, no change

Don't know

1. **Would you recommend the Anathema app to friends and/or family members?**

Yes

No

I don't know

1. **What score do you give the Anathema app?**

**In the assessment from 1 to 10 I give the following grade:**

|  |
| --- |

1. **How do you think we can improve the app?**

|  |
| --- |

**II – System Usability Scale core list with 10 questions for user-friendliness**

(System Usability Scale)

For the ten statements, indicate whether you strongly disagree, disagree, neutral, agree or strongly agree with them.

|  | Strongly disagree | Disagree | Neutral | Agree | Strongly agree |
| --- | --- | --- | --- | --- | --- |
| 1. I think I would like to use this system frequently. |  |  |  |  |  |
| 1. I found the system unnecessarily complex. |  |  |  |  |  |
| 1. I found the system was easy to use. |  |  |  |  |  |
| 1. I think that I would need the support of a technical person to be able to use this system. |  |  |  |  |  |
| 1. I found the various functions in this system were well integrated. |  |  |  |  |  |
| 1. I thought there was too much inconsistency in this system. |  |  |  |  |  |
| 1. I would imagine that most people would learn to use this system very quickly. |  |  |  |  |  |
| 1. I found the system very cumbersome to use. |  |  |  |  |  |
| 1. I felt very confident using the system. |  |  |  |  |  |
| 1. I needed to learn a lot of things before I could get going with this system. |  |  |  |  |  |

Now follows a repetition of three questions from the questionnaire that you answered prior to using the Anathema app.

**III- Sexual health**

**1. To what extent are you satisfied with your current sexual life?**

| **Very dissatisfied** |  |  |  | **Very satisfied** |
| --- | --- | --- | --- | --- |
| **□** | **□** | **□** | **□** | **□** |

**IV- Quality of life**

|  |  | Very bad | Pretty bad | Neither good nor bad | Pretty good | Very good |
| --- | --- | --- | --- | --- | --- | --- |
| 1(G1) | How would you rate your quality of life? | 1 | 2 | 3 | 4 | 5 |

|  |  | Very dissatisfied | Dissatisfied | Neither satisfied nor dissatisfied | Content | Very satisfied |
| --- | --- | --- | --- | --- | --- | --- |
| 2(G4) | How satisfied are you with your health? | 1 | 2 | 3 | 4 | 5 |
