# Supplementary material for: Evaluating the User Experience of a Smartphone-Delivered Sexual Health Promotion Program for Older Adults in the Netherlands: Single-Arm Pilot Study
Source: JMIR Hum Factors. 2024 Apr 3;11:e56206. doi: 10.2196/56206 (PMC11024746; doi:10.2196/56206)
Supplement: Multimedia Appendix 2 [file humanfactors_v11i1e56206_app2.docx]

## Appendix II – Semi-structured interview script

### Usefulness and usability of the Anathema app

1. What do you think of the app?
2. How did you experience using the app?
3. What kind of phone were you using: Android or iPhone?
4. Which part was most useful to you?
5. What were the main problems you encountered?
6. How difficult was it to solve those problems?
7. What other issues did you encounter while using the app?
8. Do you have any suggestions on how we can improve those issues?
9. Is there anything else you want the app to do or entail?
10. Did you need training or support to use the app?

### Feasibility

1. Under what circumstances and at what times did you use the app?
2. Were there any places or times when you felt uncomfortable using the app?
3. Was it difficult to make time to use the application?
4. What do you think of the structure and duration of the Anathema program (8 weeks)?
5. Would you like to tell us about your experience using the app on your phone?
6. How helpful did you find module 1 on sexual health?
7. How useful did you find module 2 on sexual health and ageing?
8. How useful did you find module 3 on thoughts, emotions, communication and about problems?
9. How useful did you find module 4 on treatment options?
10. How useful did you find module 5 looking back and looking forward?
11. Could you have benefited from support in another by a professional (e.g. phone call, video conference, in person)?

### Clinical aspects

1. Do you believe that the expectations you had/goals you set before using the app were met or exceeded?
2. What impact did the use of Anathema have on your sexual health (e.g. knowledge or satisfaction about sexuality)?
3. What do you think of the content and exercises you have in the app?
4. How useful were the app's content and exercises to you?
5. How can the app be improved to better meet your needs?
6. Which themes do you find important to find information about in the app?

### Implementation

1. Would you recommend the Anathema program to others? If so/if not, why?
2. Assuming the issues and proposed improvements are implemented. How should the Anathema program be made available to others?
3. Is there anything else you'd like to mention that we haven't addressed yet?
